# Environmental footprints of Mediterranean versus Western dietary patterns: beyond the health benefits of the Mediterranean diet

**DOI:** 10.1186/1476-069X-12-118

**Published:** 2013-12-30

**Authors:** Sara Sáez-Almendros, Biel Obrador, Anna Bach-Faig, Lluis Serra-Majem

**Affiliations:** 1 Department of Health Sciences, Universitat Oberta de Catalunya, Roc Boronat, 117, Barcelona 08018, Spain; 2 Department of Ecology, Faculty of Biology, University of Barcelona, Diagonal 643, Barcelona 08028, Spain; 3 Mediterranean Diet Foundation, c/Johann Sebastian Bach 28, Barcelona 08021, Spain; 4 Department of Clinical Sciences, University of Las Palmas de Gran Canaria, Luis Pasteur s/n, Las Palmas de Gran Canaria 35016, Spain; 5 Ciber Fisiopatología Obesidad y Nutrición (CIBEROBN, CB06/03), Instituto de Salud Carlos III, Madrid 28029, Spain

**Keywords:** Mediterranean diet, Environmental footprints, Western pattern, Sustainable diets, Spain, Sustainability, Environment

## Abstract

**Background:**

Dietary patterns can substantially vary the resource consumption and environmental impact of a given population. Dietary changes such as the increased consumption of vegetables and reduced consumption of animal products reduce the environmental footprint and thus the use of natural resources. The adherence of a given population to the Mediterranean Dietary Pattern (MDP) through the consumption of the food proportions and composition defined in *the new Mediterranean Diet pyramid* can thus not only influence human health but also the environment. The aim of the study was to analyze the sustainability of the MDP in the context of the Spanish population in terms of greenhouse gas emissions, agricultural land use, energy consumption and water consumption. Furthermore, we aimed to compare the current Spanish diet with the Mediterranean Diet and in comparison with the western dietary pattern, exemplified by the U.S.A. food pattern, in terms of their corresponding environmental footprints.

**Methods:**

The environmental footprints of the dietary patterns studied were calculated from the dietary make-up of each dietary pattern, and specific environmental footprints of each food group. The dietary compositions were obtained from different sources, including food balance sheets and household consumption surveys. The specific environmental footprints of food groups were obtained from different available life-cycle assessments.

**Results:**

The adherence of the Spanish population to the MDP has a marked impact on all the environmental footprints studied. Increasing adherence to the MDP pattern in Spain will reduce greenhouse gas emissions (72%), land use (58%) and energy consumption (52%), and to a lower extent water consumption (33%). On the other hand, the adherence to a western dietary pattern implies an increase in all these descriptors of between 12% and 72%.

**Conclusions:**

The MDP is presented as not only a cultural model but also as a healthy and environmentally-friendly model, adherence to which, in Spain would have, a significant contribution to increasing the sustainability of food production and consumption systems in addition to the well-known benefits on public health.

## Background

The environmental consequences of food systems are on public health agendas. Foods are produced, processed, distributed and consumed, these actions having consequences for both human health and the environment [1]. Furthermore, food production is also inevitably a driver of environmental pressures, particularly in relation to climate change, water use, toxic emissions and [2] greenhouse gas emissions (GHG), such as CO_2_, CH_4_ and N_2_O, which are responsible for global warming. Agriculture is one of the main contributors to the emissions of two last gases mentioned whilst other parts of the food system contribute to carbon dioxide emissions due to the use of fossil fuels in processing, transportation, retailing, storage, and preparation. Food items differ substantially in their environmental footprints, which among many other descriptors, can be measured in terms of energy consumption, agriculture land use, water consumption or GHG emissions [3]. Animal-based foods are by far the most land and energy intensive compared to foods of vegetable origin [4]. Thus, dietary patterns can substantially vary in resource consumption and the subsequent impact on the environment, as well as on the health of a given population [3].

**Figure 1:**
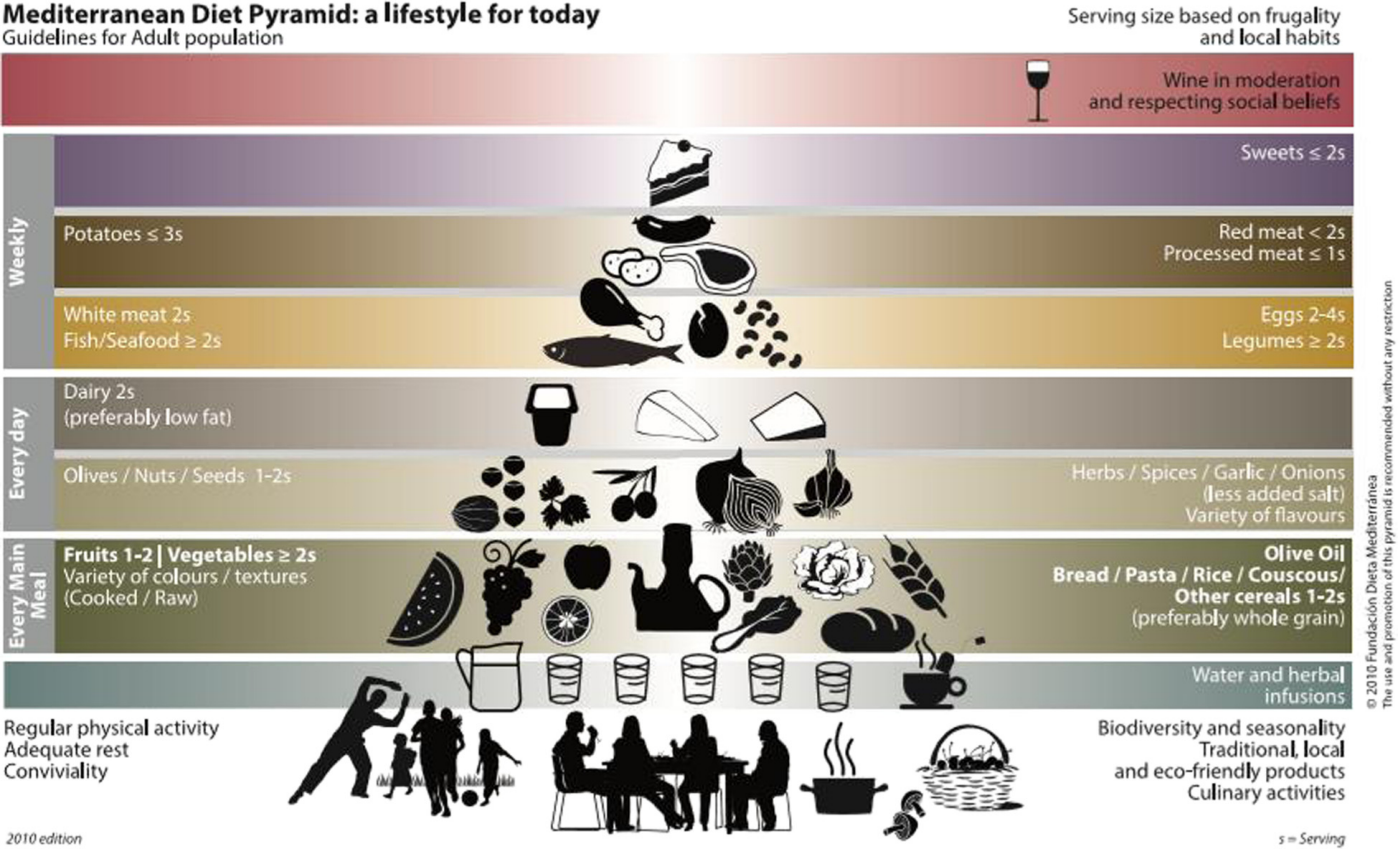
**New Mediterranean Diet pyramid.** Lifestyle guidelines for adult population. Adapted from: [11].

Research has recently shown that certain dietary patterns, such as the Mediterranean Diet (MDP), play a role in chronic diseases prevention [5]. Moreover, the MDP has been linked to a higher nutrient adequacy in epidemiological studies [6]. Thus, the MDP, as a plant-centred dietary pattern that does not exclude but rather admits moderately to low amounts of animal foods (and meat), seems to emerge as a hypothetical dietary pattern that could address both health and environmental concerns [7,8]. The MDP should be understood not only as a set of foods but also as a cultural model which involves the way foods are selected, produced, processed and distributed [9,10]. The MDP has recently been acknowledged by UNESCO as an Intangible Cultural Heritage of Humanity [9]. Based on the latest scientific evidence it has also been represented in *the new MDP pyramid*[11] (Figure 1).

Unfortunately, current diets in Mediterranean countries are departing from the traditional MDP which are changing in so far as the the quantities and proportions of food groups are concerned. This is due to the widespread dissemination of a Western-type culture, along with the globalisation of food production and consumption which is related to the homogenisation of food behaviours in the modern era [12]. The concepts of the sustainable diet and human ecology have been neglected in favour of intensification and industrialisation of agricultural systems. More recently, the growing concern over food safety has motivated a renewed interest in sustainable foods, particularly in the Mediterranean area [13].

The aim of the present study was to analyse the sustainability of the MDP in the context of the Spanish population, whilst also comparing, in terms of their environmental footprints, the current Spanish diet with both the MDP and a typical Western dietary pattern (WDP).

## Methods

Several sources of data were used to analyse the environmental footprints linked to the three dietary patterns studied. These were defined by defined by a mean consumption of the different food groups.

Dietary composition of the MDP reference pattern was obtained from the new MDP pyramid [11]. For the analysis the minimum servings of the several food groups recommended in the MDP pyramid (Figure 1) were taken into account. We assumed that the recommended servings from the MDP pyramid applied to the entire population, despite these being addressed to adult population. This limitation, and the resulting uncertainty, also apply to the other dietary patterns considered, and respond to the lack of data on specific dietary composition for different age groups. Using only the adult population would change the absolute footprints, but would in any case, only be a partial assessment of the true total footprint, and would not in fact substantially change our results concerning the relative comparison of the environmental footprints between dietary patterns.

The current Spanish dietary pattern (SCP) was estimated from the FAO food balance sheets for 2007 [14] (SCP_FB_). The WDP was exemplified by the U.S.A. food pattern, and data was also obtained from the FAO food balance sheets. This data was provided by FAOSTAT database [15]. These values reflect national per capita supply at retail level for human consumption.

At the same time, the SCP was estimated from the Household Consumption Surveys of the Spanish Ministry of Agriculture, Food and Environment [16] (SCP_CS_). The data set consisted of a representative sample of the Spanish population from 6000 households, 840 food service sector centres and 230 institutions in 2006, following a stratified random selection process which recorded daily food purchases in the first case and monthly purchases in the other two cases [16]. This allowed the estimation of the annual apparent food consumption per capita (Kg/person per year). A comparison between the two independent estimates of SCP (from food balance sheets, SCP_FB_, and from consumption surveys, SCP_CS_) was used as a quality control for the estimates. The calories from the different patterns were calculated through food composition tables and stated as comparable ranging around 2000 kcal.

The methodological limitations of the consumption surveys [17] and food balance sheets [18] should be taken into consideration when interpreting the results. Despite food purchases and food consumption being fully equivalent, mostly due to food waste that may occur at household level, the amount of food purchased reasonably corresponds to that consumed [19]. Furthermore data on foods available for human consumption obtained from the food balance sheets, generally overestimate food consumption compared to individual dietary surveys [20].

Footprints analysed include GHG emissions and use of resources such as agricultural land use, energy consumption and water consumption. Specific footprints for each food group were obtained from several Life Cycle Assessment (LCA) sources conducted in Spain and elsewhere (Additional file 1). The three phases analyzed in the food system were the agricultural production, processing and packaging, transportation and retail. These three phases were considered key, where information was available. Some specific footprint values for water consumption and GHG emissions, were not available for some of the food groups (cereals, fruits, vegetables, vegetable oils, nuts, etc.). This occurred mainly in the area of processing and transportation. The uncertainties associated with the lack of data for specific footprint values and with the assumption of constant footprints in general imply that the environmental footprint estimates discussed here should be considered as conservative.

Regarding the limitations linked to environmental food data; there were a limited number of food items included in the analysis as data on the different processes was lacking for some food-items. It was assumed that each food category was represented by some representative food items. Furthermore, post retailing (distribution from stores to households, storing and cooking) and alternative ways of production (eco-friendly) were not taken into account, only conventional agriculture processes were included. Thus, neither the environmental impact of intensive resource-consuming processed foods, nor the energy savings related to a higher contribution by fresh, local, eco-friendly and seasonal products could be evaluated in the study. Land use included both the land used for crops and livestock production, implying an inherent bias in our estimates of this environmental pressure. In the case of fish, data on land use were not available. Vegetable oils and animal fats had to be considered in one single category, which makes it difficult to obtain a clear idea of the relative contributions of “vegetal-source foods” and “animal-source foods” to different environmental footprints.

For comparison, the level of current real environmental pressure was estimated. The current footprint for each environmental pressure was taken into consideration. Thus, current land use was defined as the agricultural area, including cultivated (arable and permanent crops) and pasture areas. Data was obtained from the FAOSTAT database in 2008 [15]. Current energy consumption was estimated using data on the energy consumed by the agricultural, fishing and food-production sectors for 2009 [21,22]. Current water consumption was defined as the total amount of water consumed by the agricultural sector in 2009 and by food industries in 2008 [23]. The current GHG emissions correspond to the total gas emissions of agricultural and food industries (in grams of CO_2_ equivalent).

## Results

The MDP showed the lowest footprints in all the environmental pressures taken into consideration, whereas the WDP showed the highest (Table 1). The footprint estimates for the SCP showed considerable differences when evaluated using food balance sheets and consumption surveys estimates, the former always being higher than the latter. The Land Use and Water Consumption footprint estimates for the SCP agreed with the current real environmental pressures, i.e. the current real pressure fell between the SCP_FB_ and SCP_CS_ values. However, the Energy Consumption and GHG emissions footprint estimates for the SCP were higher than the current real pressures for both SCP_FB_ and SCP_CS_.

**Table 1:** Environmental footprints for MDP, WDP and SCP, and current real pressure for each footprint

	**MDP**	**SCP**_**FB**_	**SCP**_**CS**_	**WDP**	***Current real pressure***
**Agricultural land use (10**^**3**^**Ha year**^**-1**^**)**	8 365	19 874	12 342	33 162	15 400
**Energy consumption (TJ year**^**-1**^**)**	239 042	493 829	285 968	611 314	229 178
**Water consumption (Km**^**3**^**year**^**-1**^**)**	13.2	19.7	13.4	22.0	19.4
**Greenhouse gas emissions (Gg CO**_**2-eq**_**year**^**-1**^**)**	35 510	125 913	72 758	217 128	62 389

The subscripts FB and CS refer to estimates derived from food balance sheets and from consumption surveys, respectively.

**Figure 2:**
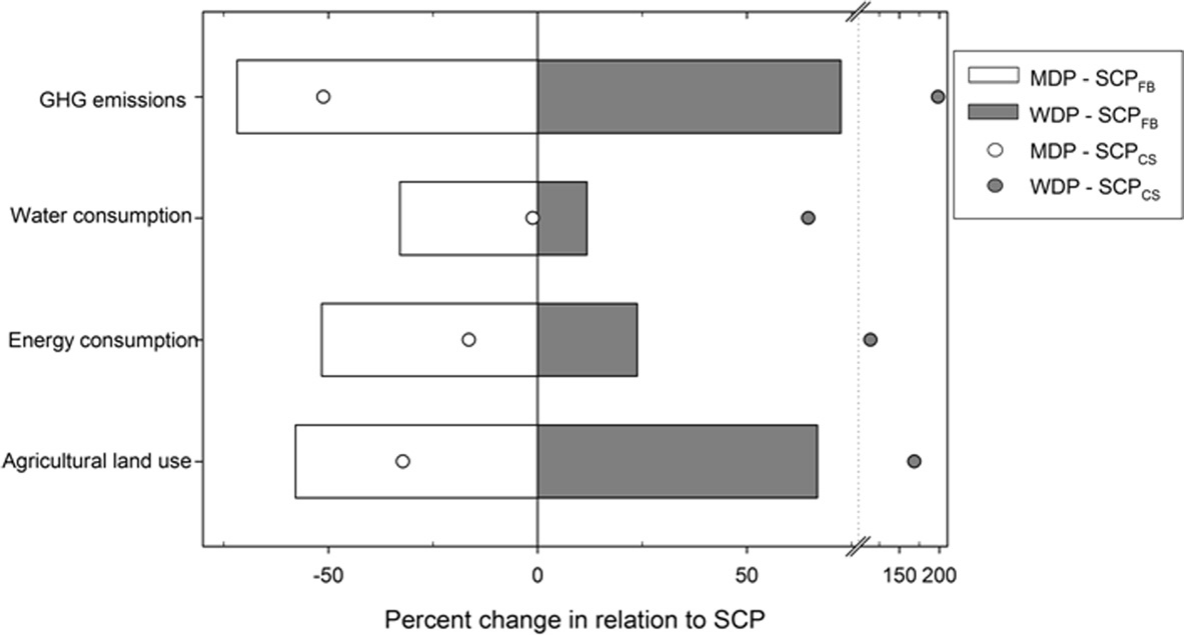
**Changes in environmental footprints of the Mediterranean (white) and Western (grey) dietary patterns in relation to the Spanish current diet.** The relative change of each dietary pattern in relation to the Spanish current diet is shown for data derived from food-balance sheets (boxes) and from household consumption surveys (dots).

The adherence of the Spanish population to the MDP would decrease all the considered environmental footprints (Figure 2). The MDP in Spain would substantially reduce GHG emissions (72%), agricultural land use (58%) and energy consumption (52%), and to a lower extent water consumption (33%). On the contrary, the adherence to a WDP would imply an increase in all of these descriptors of between 12% and 72%. Almost identical results appear if the consumption surveys’ estimates (SCP_CS_) are used. In this case, the decreases in environmental footprints for the MDP (Figure 2) are smaller: between 16% and 52% for land use, energy consumption and GHG emissions, and almost negligible for water consumption (1.2%). For the WDP using the same data sources all footprints show marked increases of between 65% and 198%.

Animal products contributed significantly to increasing diet patterns footprints. Therefore, diet patterns such as WDP and SCP, with a high contribution of animal products such as meat and dairy products present higher footprint values.

**Figure 3:**
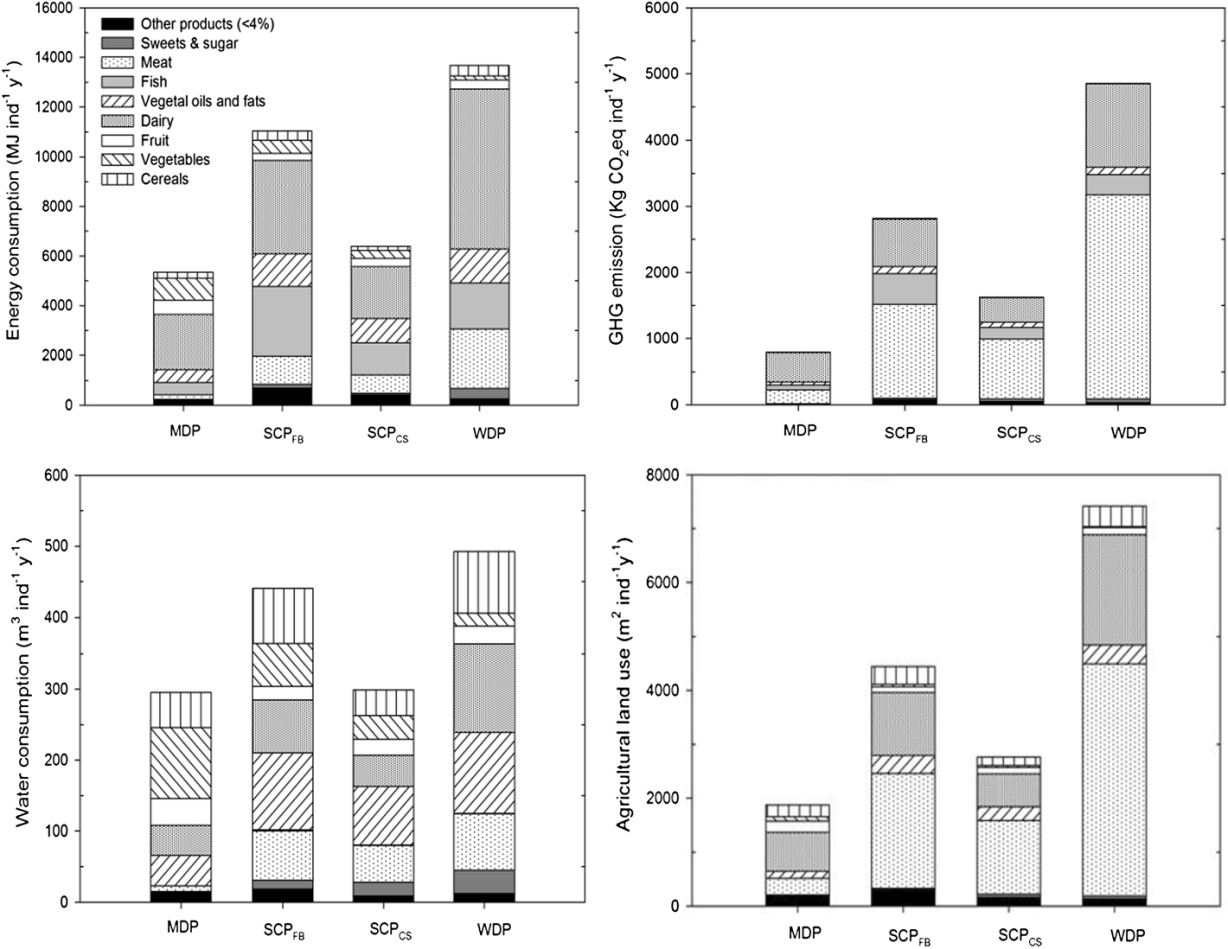
**Environmental footprints (energy consumption, water consumption, GHG emissions and agricultural land use) mean annual contribution of each food group to the dietary pattern.** *The fish group was not considered in water footprint and land use footprint because was not applicable.

The food products with the highest contribution to energy intake are dairy products followed by meat for the WDP, fish for the SCP and vegetables for the MDP (Figure 3).

Food groups that showed the highest water consumption/use are dairy and vegetable oils in a similar magnitude. In the WDP dairy products have a slightly higher contribution to water use and as do vegetable oils in the case of the MDP and SCP. Meat is in the third place for water use in all dietary patterns except in the MDP where other products have a higher contribution (especially nuts (12 440 L/capita • year) and eggs (2 430 L/capita • year). In the WDP sugars and sweets occupy the fourth place in contribution to water use (Figure 3).

Regarding GHG emissions, undoubtedly meat stands as the food item that most contributes to emissions; a large difference compared to other foods emerges, both in the WDP and SPC. However, dairy products are the main contributor to GHG emissions in the MDP. In second place, we find dairy products in both the WDP and SCP, and meat in the MDP. In third place, comes fish in all the dietary patterns (Figure 3). A WDP would account for double the GHG emissions compared with the SCP whilst WDP would produce 6 times greater emissions than the MDP.

As in the case of GHG emissions, meat is the food that most contributes to land use in both the WDP and the SCP with high values of m^2^/capita and showing considerable differences in comparison with other food groups. Meanwhile dairy products in the MDP show the highest contribution to land use. In the WDP and SCP meat is followed by dairy products and for the MDP, dairy products are followed by meat and then, cereals and vegetable oils (Figure 3).

Regarding the environmental footprint mean annual contribution of each food group to the dietary pattern; we observed that in the MDP, vegetables, fruit, and to a lesser extent cereals and vegetable oils have greater weight, and had a comparatively higher contribution to water consumption and, to a lesser extent, energy consumption. Dairy products, as one of the main sources of animal protein in the MDP, were the food group which presented the highest footprint in all four analyzed footprints (Figure 3).

Environmental footprints of food groups means were found to be similar in the WDP and in the SCP in a lower weight but with similar relative contributions. Although, in both patterns, dairy, fish and vegetable oils were foods that contributed substantially in terms of energy consumption, in the WDP there was clearly a higher contribution of meats and dairy food groups (Additional file 2). On the other hand, in the SCP the energy contribution for vegetables was far more relevant. In these patterns, in the case of food related water consumption, vegetable oils followed by dairy and meats were the foods that contributed most. However, in the WDP sweets had a greater weight. In terms of GHG emissions and land use in both dietary patterns, meat, followed by dairy were the most contributing food. Meat figured in a much higher proportion.

## Discussion

Studies such as the present on the assessment of food related environmental impacts of mean food dietary patterns, generally conclude that a shift towards less animal-based and more plant-based diets would have both a beneficial effect on climate and on the environment overall [3,4,24-29]. In the present study, it was found that the MDP implies lower demands on soil, compared to both the SCP and to WDP, and also on water and energy resources (even though our estimates were conservative). In fact, it was observed that a shift towards MDP would result in a reduction of the Spanish environmental footprint in any of the considered pressures from 33 to 72%. On the contrary, a progressive shift towards WDP would imply an increase in the footprints (12-72%). These results reinforce the sustainable character of the MDP in an increasingly globalised world [30,31].

The comparison of the results on food environmental footprints between studies is complex because the results depend on a large variety of factors. A major limitation of this study was the use of data that has not been recently published, and the use of data from different countries (e.g. Northern Europe) belonging to different agro-ecological zones. However, despite some aforementioned limitations related to food intake and to environmental data sources used, the results in the present study are in line with most of the available literature [30,31].

We consider our footprint estimates for land use and water consumption for the SCP to be realistic due to the agreement with the current real environmental pressures (Table 1). On the contrary, a slight overestimation of the energy consumption and GHG emissions footprints is acknowledged for the SCP. A comparison of the environmental footprints of the MDP and the WDP with the current real environmental pressures (rather than with the SCP) shows that the WDP would substantially increase all the environmental footprints. The adherence of the Spanish population to the MDP would decrease all the environmental pressures except energy consumption which would show an almost imperceptible increase.

As far as the environmental contribution of the different food groups is concerned, most of the literature available, despite originating from different settings and types of analysis, converges overall in the global statements. Plant-based foods were the group that contributed least to the selected environmental footprint, and as expected, in the MDP meat and dairy presented lower figures of water consumption and to a lesser extent energy consumption compared to the other patterns. Plant foods based on vegetables, cereals, and legumes are notably the food group with the lowest GHG emissions even where processing and substantial transportation is involved [3]. In our study, legumes were included in the vegetable group as was indicated in other studies and since separate LCA data was not available. In fact, legumes are stated as alternatives to animal protein foods due to their low environmental impact and long durability [3]. However, some vegetable origin foods contribute substantially, together with dairy products, in the case of the MDP and SCP, to either water consumption (vegetable oils in particular, and to some extent nuts) or land use (cereals and vegetable oils) in their production. In both the SCP and in the WDP, vegetal oils also contributed to a great extent to water consumption and to energy consumption footprints. Meanwhile animal-based foods were found to cause the highest environmental impact in all dietary patterns. As in other studies of the Spanish context, meat and dairy were the foods that most contributed to environmental footprints [32], although at a much lower absolute contribution than the WDP. As far as GHG emissions and land use were concerned, undoubtedly meat resulted as the food item that contributed most, showing a large difference compared to other foods, both in the WDP and SCP. It was observed that a reduction in meat consumption decreased GHG emissions [31], and land use, subsequently increasing the availability of land for other uses [31]. Even though there is high production variability [3] which may be as much of 80% of global agriculture across countries, land use is related to livestock production and accounts for more than half of the GHG emissions resulting from agriculture [14]. Meanwhile, dairy products, one of the main sources of animal protein in the MDP, contributed to a great extent in terms of energy consumption in the three dietary patterns. In the MDP, dairy products was the food group which presented the highest footprint in all 4 analyzed footprints since in the MDP meat has a lower weight compared to the other patterns both in frequency and amount (Additional file 3) [11]. Regarding GHG emissions; fish also showed a remarkable environmental contribution in all the dietary patterns. According to our results, the MDP in Spain would substantially reduce overall water consumption: despite a possible increase in water consumption from vegetables and fruit groups. Water consumption of certain food groups as vegetable oils and fats or meat products would be lower than the WDP.

The most relevant dietary distinctions in terms of environmental cost were those that occurred between animal-based versus plant-based diets, with an important influence of the various ways foods are grown, processed, and transported. The largest environmental impact of food production from the farm level to consumers is generally associated with primary production. In terms of energy consumption, differences in greenhouse production versus open-air cultivation of certain crops, and canned or frozen-produce versus fresh-produce are substantial [25,33]. Besides the energy involved in agricultural production, the amount of energy used in household food storage, preparation and waste is not negligible [3].

Food policy and dietary guidelines need to develop and move on from the classical approach which only focuses on nutrients and health, to one that takes into consideration environmental impact. Even consumers are tending to become more and more concerned about the environment and, even more so, about their personal health, there is a strong resistance to changing certain food choices (i.e. reducing meat consumption) whilst cultural culinary traditions are not easy to modify. Some studies state that even radical changes in food consumption patterns would provoke quite small environmental benefits [27-29]. A significant reduction in environmental footprints through a shift from the SCP towards a MDP type diet, would probably not only require substantial changes in consumers’ food choices but more specifically changes in agro-food-industry practices, public catering supply and agricultural and trade policies [7,29,34,35]. Spain is one of the major producers and exporters of typical Mediterranean products, thus it would make sense to maintain a MDP agricultural production model.

## Conclusion

A shift from the current Spanish pattern towards the Mediterranean dietary pattern through the use of the *new Mediterranean Diet pyramid*[11] would be beneficial from both a health and environmental perspective. The Mediterranean dietary pattern presented lower footprints than the current Spanish pattern, and to a much larger extent than the Western dietary pattern. The Mediterranean dietary pattern results in a lower environmental impact due to the consumption of more plant-derived products and less animal products. The Mediterranean dietary pattern is presented as not only a cultural model but as a healthy and environmentally friendly model, adherence to which in Spain would make a significant contribution to greater sustainability of food production and consumption, in addition to the well-known benefits on public health.

## Abbreviations

MDP: Mediterranean diet; GHG: Greenhouse gases; CO2: Carbon dioxide; CH4: Methane; N2O: Nitrous oxide; UNESCO: United Nations Educational Scientific and Cultural Organization; WDP: Western dietary pattern; SCP: Spanish current dietary pattern; SCPFB: SCP estimated from food balance sheets; SCPCS: SCP estimated from the household consumption surveys: Life Cycle Assessment (LCA).

## Competing interests

The authors have no conflict of interest to declare.

## Authors’ contributions

All authors have made substantial contributions to conception and design, or acquisition of data, or analysis and interpretation of data; SSA and ABF have been involved in drafting the manuscript; and all authors have been revising it critically for important intellectual content, given final approval of the version to be published. LSM acts as a guarantor of the content of this paper.

## Supplementary Material

Additional file 1 Environmental footprint sources of references (citation, geographic area and year) for the different production and distribution phases. Click here for file

Additional file 2 Relative intakes of each food group to the MDP and relative contributions to environmental footprints. Click here for file

Additional file 3 Composition of the dietary patterns considered (Kg ind-1 y-1). Click here for file
