# Supplementary material for: Environmental footprints of Mediterranean versus Western dietary patterns: beyond the health benefits of the Mediterranean diet
Source: Environ Health. 2013 Dec 30;12:118. doi: 10.1186/1476-069X-12-118 (PMC3895675; doi:10.1186/1476-069X-12-118)
Supplement: Additional file 1 — Environmental footprint sources of references (citation, geographic area and year) for the different production and distribution phases. [file 1476-069X-12-118-S1.pdf]

**Additional file 1. Environmental footprint sources of references (citation, geographic area and year) for the different production and distribution phases**

| | Food group | LCA Phases | | |
|---|---|---|---|---|
| | | Agricultural production | Processing and packaging | Transportation and retail |
| **Agricultural land use** | Cereals, potatoes, vegetables, fruit, nuts and vegetable oils | Spain 2009[1] | not applicable | |
| | Dairies | Sweden 1997[2] | | |
| | Eggs | Denmark 2000-2002[3,4] | | |
| | meat and fish | Denmark 2000-2002[4] | | |
| | Wine | Spain 2009[1,5] | | |
| | sweets and sugar | Spain 2009 [1,4] | | |
| **Energy consumption** | Cereals (except bread) and legumes | Spain 2008[3] | World average[6] | World average[6] |
| | bread and sugar | Denmark 2000-2002[4] | | |

| | | | | |
|---|---|---|---|---|
| | potatoes and sweet potatoes | United Kingdom 2006[7] | | |
| | Vegetables | Spain 2008[3] (Data for tomatoes) | United Kingdom 2006[7] | |
| | Fruit | Spain 2008[3] | United Kingdom 2006[7] | |
| | Dairies | Europe 1990-2008[8] | | |
| | Nuts | Cyprus 2000-2007[9] | World Average[6] | World Average[6] |
| | eggs and meat | Denmark 2000-2002[3,4] | | |
| | Fish | Denmark 2000-2002[4] | Spain 2004[10] | World Average[6] |
| | vegetable oils | Cyprus 2000-2007[9] | | World Average[6] |
| | Wine | Spain 2005[11] | | |
| | Sweets | Spain 2008 and Denmark 2000-2002[3,4] | Denmark 2000-2002[4] | |
| **Water consumption** | Cereals (except bread) and fruit | Spain 2008[3] | n. a. | n. a. |
| | Bread, potatoes, nuts, sweets and sugar | World Average 1996-2005[12] | | |
| | Legumes | World Average. Data for beans 1996-2005[12] | | |
| | Vegetables | Spain 2008[3] (Data for tomatoes) | n. a. | n. a. |
| | Dairies, eggs and meat | Spain 1996-2005[12] | | |

| | | | | |
|---|---|---|---|---|
| | Fish | Spain 2004 and Denmark 2000-2002[4,13] | Spain 2004[10] | n. a. |
| | Vegetable oils | Cyprus 2000-2007[9] | | n. a. |
| | Wine | Spain 2005[11] | | |
| **GHG emissions** | cereals (except bread), fruit | Spain 2008[3] | n. a. | n. a. |
| | bread, potatoes and sweet potatoes and sugar | United Kingdom 2006[7] | | |
| | Legumes | United Kingdom 2006[7] (Data for beans) | | |
| | Vegetables | Spain 2008[3] (Data for tomatoes) | n. a. | n. a. |
| | Dairies | Europe 1990-2008[8] | | |
| | Nuts | Cyprus 2000-2007[9] | n. a. | n. a. |
| | Eggs | Spain 2007[3] | n. a. | n. a. |
| | meat (white and pork) | Spain 2007[3] | Denmark 2000-2002[4] | |
| | meat (cattle and fats) and fish | Denmark 2000-2002[4] | | |
| | Olive oil | Cyprus 2000-2007[9] | | n. a. |
| | Wine | Spain 2005[11] | | |

| | | |
|---|---|---|
| | Sweets | United Kingdom 2006[7] (Data for cookies) |

n. a. : not available

---

[1] Food and Agriculture Organization of the United Nations: **FAOSTAT** [http://faostat.fao.org/].

[2] Cederberg C, Mattsson B: **Lifecycle assessment of milk production – a comparison of conventional and organic farming.** *Journal of Cleaner Production* 2000, **8**:49-60.

[3] Garrido A, Bardají I, De Blas C, García R, Hernández Díaz-Ambrona C, Linares P: Indicadores de sostenibilidad de la agricultura y ganadería españolas (Spanish agriculture and livestock indicators of sustainability). Plataforma Tecnológica de Agricultura Sostenible (Sustainable Agriculture Technology Platform). Escuela técnica superior de ingenieros agrónomos (Higher Technical School of Agricultural Engineers). Madrid: Universidad Politécnica de Madrid (Polytechnic University of Madrid); 2011.

[4] **LCA food data base** [www.lcafood.dk]

[5] **European Comission database Eurostat** [http://epp.eurostat.ec.europa.eu/portal/page/portal/statistics/themes].

[6] Heller M, Keoleian G: **Life-Cycle Based Sustainability Indicators for Assessment of the U.S. Food System**. Ann Arbor: University of Michigan; 2000.

[7] Foster C, Green K, Bleda M, Dewick P, Evans B, Flynn A, Mylan J: **Environmental Impacts of food production and consumption. A report to the Department for environment, food and rural affair.** London: Manchester Business School and DEFRA; 2006.

[8] International Dairy Federation. Environmental/Ecological Impact of the dairy sector: Literature review on dairy products for an inventory of key issues. List of environmental initiatives and influences on the dairy sector. Bulletin of the IDF 2009; Nº 436.

[9] Avraamides M, Fatta D: **Resource consumption and emissions from olive oil production: a life cycle inventory case study in Cyprus.** *Journal of cleaner production* 2008, **16**:809-821.

[10] MAFE (Ministry of Agriculture, Food and Environment, Spain): La alimentación en España 2006 (Food in Spain 2006). Madrid; 2007.

[11] Gazulla C, Raugei M, Fullana-i-Palmer P: **Taking a life cycle look at crianza wine production in Spain: where are the bottlenecks?**. *International Journal Life Cycle Assessment* 2010, **15**:330-337

[12] Mekonnen MM, Hoekstra AY: **The green, blue and grey water footprint of crops ans derived crop products. Hydrology and Earth System Sciences.** *Hydrol Earth Syst Sci* 2011, 15:1577–1600. [http://www.waterfootprint.org/?page=files/WaterStat-ProductWaterFootprints].

[13] Ministerio de Medio Ambiente y Medio Rural y Marino: (Ministry of environment, rural and marine, Spain). Guía de Mejores Técnicas Disponibles en España del sector de Productos del mar (Best Available Techniques Guide of Sea products sector in Spain). Spain; 2006.
