# Supplementary material for: Environmental footprints of Mediterranean versus Western dietary patterns: beyond the health benefits of the Mediterranean diet
Source: Environ Health. 2013 Dec 30;12:118. doi: 10.1186/1476-069X-12-118 (PMC3895675; doi:10.1186/1476-069X-12-118)
Supplement: Additional file 2 — Relative intakes of each food group to the MDP and relative contributions to environmental footprints. [file 1476-069X-12-118-S2.pdf]

Additional file 2. Relative intakes of each food group to the MDP and relative contributions to environmental footprints.

		Relative contribution to total footprints			
**Food group**	**Contribution to the MDP intake**	**Land use**	**Energy consumption**	**Water consumption**	**Greenhouse gas emissions**
Cereals	11%	12%	4%	17%	0%
Vegetables	41%	4%	17%	34%	1%
Fruit	27%	11%	11%	13%	0%
Dairy products	11%	38%	41%	14%	55%
Vegetal oils and fats	2%	7%	10%	14%	5%
Fish	2%	0%	9%	0%	9%
Meat	2%	16%	4%	3%	27%
Sweets & sugar	<1%	0%	0%	0%	0%
Other products	4%	11%	4%	5%	2%
