# Supplementary material for: Environmental footprints of Mediterranean versus Western dietary patterns: beyond the health benefits of the Mediterranean diet
Source: Environ Health. 2013 Dec 30;12:118. doi: 10.1186/1476-069X-12-118 (PMC3895675; doi:10.1186/1476-069X-12-118)
Supplement: Additional file 3 — Composition of the dietary patterns considered (Kg ind-1 y-1). [file 1476-069X-12-118-S3.pdf]

Additional file 3. Composition of the dietary patterns considered (Kg ind^{-1} y^{-1})

**Food group**	**MDP**	**SCP**	**WDP**
Cereals	75	98	112
Vegetables	269	160	49
Fruit	175	88	111
Dairy products	71	178	254
Vegetal oils and fats	11	28	29
Fish	14	81	54
Meat	16	117	133
Sweets & sugar	<1%	26	68
Other products (<4%)	25	111	83
